# Space travel has effects on planarian regeneration that cannot be explained by a null hypothesis

**DOI:** 10.1002/reg2.89

**Published:** 2017-12-05

**Authors:** Michael Levin, Junji Morokuma, Joshua Finkelstein

**Affiliations:** ^1^ Allen Discovery Center Tufts University 200 Boston Ave Medford MA 02155–4243 USA

We thank the editors of *Regeneration* for the opportunity to respond to the letter by Sluys and Stocchino (2017) (S&S), who take issue with our report of observations (Morokuma, Durant, & Williams, 2017) on planaria that spent several weeks aboard the International Space Station (ISS), in comparison with controls that stayed (similarly sealed) on Earth.

First, we give a brief review of what we did and did not claim in the original study. Our paper describes what we observed in the “space‐exposed” animals upon return to Earth. We saw significant differences in behavior, water metabolite content, and microbiome composition. Moreover, one of the animals came back as a biaxial heteromorphosis (having heads on both ends of the main body axis). We did not claim to have determined which of the many aspects of the space travel experience (loss of gravitational field, reduced geomagnetic field, effects of high G‐force or vibration during take‐off and splashdown, etc.) induced these marked changes, nor did we claim to have identified the molecular mechanism by which the changes were induced. We were clear that this is (necessarily, given the logistics of space flight) a small pilot experiment and that many future experiments will be necessary to mechanistically understand the processes by which space travel interacts with biological systems. At the same time, our study reveals clear, statistically significant differences between space‐exposed and Earth‐bound controls, which cannot be swept under the rug without rigorous argument.

We now summarize the facts regarding the double‐headed worm phenotype, which is the focus of S&S's critique. As far as we can tell, the argument by S&S is as stated at the end of their Abstract: “Double‐headed worms have been amply documented as arising under experimental conditions as well as spontaneously in stock cultures of planarians.” The first part is a *non‐sequitur*: certainly there are other experimental treatments that can cause the same phenotypeour laboratory showed that treating *Dugesia japonica* with gap junction blockers generates double‐headed worms (Nogi & Levin, 2005; Oviedo, Morokuma, & Walentek, 2010). As we hope is clear from the text of our paper, we never claimed space travel to be the *only* way to induce double‐headed worms or that double‐headed worms had never been observed before. Regardless of the fact that a few other treatments can also induce this phenotype, such treatments were not present on the ISS and are quite irrelevant here. Moreover, the claim that double‐headed worms arise spontaneously in stock cultures is misleading. Whilst a double‐headed worm could form spontaneously, this is an extremely rare event; surely S&S are not suggesting that, in their *D. japonica* stock colony, one out of 15 cut worms becomes double‐headed (as happened in our space‐exposed worms)? If not, then their point is moot. If yes, then this suggests strikingly suboptimal culture conditions in their own planarian cultures, and certainly does not reflect the experience of the rest of the flatworm community. Planaria under normal conditions of stock culture throughout the world (including the actual controls we reported in our study) do not produce double‐headed forms at anywhere near a frequency of 1/15. (It should parenthetically be noted that the correct denominator is 15, not 30 as stated by S&S.) Next, we examine some of the detailed claims in S&S's letter.

(1) “[The claim that] the occurrence of such heteromorphoses is extremely rare are unjustified and mostly rest on an eclectic survey of the literature.” Our analysis of the background rates of double‐headed worms are not based on a survey of literature: it is based on our internal controls and the historical controls of many thousands of *D. japonica* worms observed in our laboratory (including middle‐third cut experiments) for the last 17 years. In order to argue that this is a statistical fluke, S&S would have to cite data suggesting a rate of ∼1 in 15 two‐headed outcomes in cut *D. japonica*. Instead, they cite a number of very old papers in species like *Procerodes lobatus*, *Cercyra hastata*, *Girardia dorotocephala*, *G. schubarti*, *G. tigrina*, and *Cura foremanii* (about all of which our paper made no claims). The only sources of information about *D. japonica* to which they refer are Brøndsted (1969) and Ichikawa and Kawakatsu (1964), and neither contains hard numbers on spontaneous rates of double‐headed worms in cultures or other experiments. As S&S themselves point out, “spontaneous, natural occurrence of bipolar worms has been observed much more rarely in stock cultures of planarians”; the rate would have to be similar to what we saw in the space‐exposed worms to allow them to draw a statistically valid conclusion about our findings being due to chance. As just one comparison, a paper cited by S&S (Kanatani, 1958) reports zero (0) double‐headed worms out of 1186 fragments cut. In any case, rather than relying on very old literature done in different species (and often not reporting specific statistics), we invite S&S to culture *D. japonica* under standard conditions and publish the rate of spontaneous double‐headed worms from carefully done amputation experiments. We predict that it will be several orders of magnitude lower than 1/15.
(2) “[E]xperimentally induced double‐headedness in planarians has been amply documented”; this is true, but does not imply anything about the origin of our double‐headed phenotype. The fact that other treatments, which S&S correctly identify (e.g., gap junction inhibitors [Oviedo et al., 2010] and cytoskeletal disruptors [Kanatani, 1958]), cause double‐headedness is irrelevant here because (a) we do not claim space travel to be the *only* source of bipolarity, and (b) there was no octanol or any other small molecules/proteins applied during their ISS sojourn. The worms were cut on Earth and immediately placed in Poland Spring water; the tubes were not re‐opened until they returned to our laboratory, several weeks later. S&S's reminder of the effects of β‐catenin on *Schmidtea mediterranea* is likewise irrelevant — surely they are not suggesting that β‐catenin was somehow introduced into the worms on the ISS? The fact is that worm regeneration is very robust; it requires significant effort via a specific reagent to induce bipolarity at an observable rate, and those reagents were not used in our experiments. And if space travel can mimic the mechanisms of gap junctional inhibition or β‐catenin pathway modulation, this is a result well worth knowing.
(3) S&S's position on the permanence of the double‐headed form is unclear. They refer to it as “*presumed* stable” (suggesting that they do not believe our report of several subsequent regeneration events which exhibited two heads) but in the same paragraph call it “unsurprising,” which suggests that it is not only believable but expected. Actually, the double‐head permanence is neither inevitable nor impossible, as we have conditions (unpublished) in our experimental work that produce double‐headed worms that do not become double‐headed in subsequent cuts.
(4) S&S refer to a single bipolar animal in an unidentified species from Liguria, Italy (referencing a poster). Unfortunately this anecdote lacks the side‐by‐side controls that our study reported. We have no idea to what influences the Ligurian worm had been exposed or how that species’ regenerative mechanisms relate to *D. japonica* used in our study. In contrast, our study had very strict protocols guaranteeing that the experience of space travel was the major difference between our controls and experimental populations.
(5) “[T]here is yet no indication that the regenerative processes differ among species of planarians” — this is quite untrue. Not only have there been many reports (both classical [Brøndsted, 1969] and recent [Umesono, Tasaki, & Nishimura, 2013]) about significant native differences in regenerative ability and other outcomes among planaria, we (and the planarian community as a whole) are well aware of the considerable differences in response of, for example, *D. japonica* and *S. mediterranea*, to drugs, RNAi, and various environmental stressors. As just another example, octanol causes bipolarity in *D. japonica* (Durant et al., 2017), but changes of head shape in *Girardia dorotocephala* (Emmons‐Bell, Durant, & Hammelman, 2015).
(6) “[R]eversed polarity during reproduction or regeneration occurs also in the flatworm‐like metazoans *Convolutriba retrogemma* and *C. macropyga*. In these species asexual reproduction involves a budding process in which buds are generated with a body axis orientation that is the reverse of that of the parent animal.” We thank S&S for the pointer to this very interesting work; its direct relevance for our study is not obvious.
(7) “[E]xposure of the pharyngeal pieces of the worms to conditions in the International Space Station started only approximately 78 h after amputation and that thus regenerative processes were already well in progress.” S&S may be unaware that, even 3 days after amputation, regenerative patterning decisions can be over‐ridden (Durant et al., 2017, Fig. 2).
(8) “[L]ook first for Earth‐bound processes and determinants.” This is good advice indeed, and we had done so, in the planning of our study, in the execution of careful controls, and in the analysis afterwards. Given the lack of octanol, β‐catenin, colchicine, etc. in the container in which the planaria went to space, we welcome any specific suggestions from S&S as to what factor, other than the lengthy and eventful experience of travel to and from space, better represents a likely reason for the many changes we observed. We note parenthetically that S&S do not suggest the other differences we observed as likewise being due to chance. If microbiome, metabolism, and behavior can all plausibly be altered by space travel, it is hard to see why the complex process of anterior−posterior patterning cannot be.

In conclusion, we welcome interest in our data but cannot see any substantive arguments in S&S's letter that could convincingly lead to different conclusions than the minimalist interpretation we offered for our observed findings. Indeed, their title “Bipolarity in planarians is not induced by space travel” is a strong positive claim that needs proof; the only way that such a claim can be supported is with actual data: repeat experiments showing that habitation on the ISS had no effect on *D. japonica*. It is of course true that future work is necessary to follow up our limited pilot study; additional differences due to space travel and also previously unrecognized factors feeding into cellular decision‐making during regeneration no doubt remain to be uncovered. We encourage S&S (and anyone else) to contribute to this fascinating field by continuing such experiments and learning more about the effects of space travel. Unless contradictory data become available from future experiments (as past papers offer no sign of higher background rates of double‐headedness in control populations), it is not reasonable to claim definitive *lack* of effects of space travel on regeneration counter to our observations. We thank S&S for the opportunity to highlight these important points, and stand by the original findings: in our experiment, exposure to space travel, compared to Earth‐bound controls, clearly had effects upon planaria, including the induction of a biaxial form that cannot be explained by the null hypothesis.
